# Trends in Acute Myocardial Infarction Hospitalization Rates for US States in the CDC Tracking Network

**DOI:** 10.1371/journal.pone.0064457

**Published:** 2013-05-22

**Authors:** Evelyn O. Talbott, Judith R. Rager, LuAnn L. Brink, Stacey M. Benson, Richard A. Bilonick, Wen Chi Wu, Yueh-Ying Han

**Affiliations:** 1 University of Pittsburgh, Graduate School of Public Health, Department of Epidemiology, Pittsburgh, Pennsylvania, United States of America; 2 University of Pittsburgh, School of Medicine, Department of Ophthalmology, Pittsburgh, Pennsylvania, United States of America; 3 University of Pittsburgh, Graduate School of Public Health, Department of Biostatistics, Pittsburgh, Pennsylvania, United States of America; 4 Division of Pediatric Pulmonary Medicine, Allergy, and Immunology, Children's Hospital of Pittsburgh of UPMC, University of Pittsburgh, Pittsburgh, Pennsylvania, United States of America; Fundación para la Prevención y el Control de las Enfermedades Crónicas No Transmisibles en América Latina (FunPRECAL), Argentina

## Abstract

**Objectives:**

We examined temporal trends, spatial variation, and gender differences in rates of hospitalization due to acute myocardial infarction.

**Methods:**

We used data from the Centers for Disease Control National Environmental Public Health Tracking Network to evaluate temporal trends, geographic variation, and gender differences in 20 Environmental Public Health Tracking Network states from 2000 to 2008. A longitudinal linear mixed effects model was fitted to the acute myocardial infarction hospitalization rates for the states and counties within each state to examine the overall temporal trend.

**Results:**

There was a significant overall decrease in age-adjusted acute myocardial infarction hospitalization rates between 2000 and 2008, with most states showing over a 20% decline during the period. The ratio of male/female rates for acute myocardial infarction hospitalization rates remained relatively consistent over time, approximately two-fold higher in men compared to women. A large geographic variability was found for age-adjusted acute myocardial infarction hospitalization rates, with the highest rates found in the Northeastern states. Results of two ecological analyses revealed that the NE region remained significantly associated with increased AMI hospitalization rates after adjustment for socio-demographic factors.

**Conclusions:**

This investigation is one of the first to explore geographic differences in AMI age adjusted hospital rates in individuals 35+ years of age for 2000–2008. We showed a decreasing trend in AMI hospitalization rates in men and women. A large geographic variability in rates was found with particularly higher rates in the New England/Mid-Atlantic region of the US and lower rates in the mountain and Pacific states of the tracking network. It appeared that over time this disparity in rates became less notable.

## Introduction

Since 2002, the Centers for Disease Control and Prevention (CDC) has been building the infrastructure to create, organize and maintain a national data network which will permit the collection, use and dissemination of nationally consistent information to mount an effective national response to environmentally mediated acute and chronic disease “outbreaks.” To further develop the National Environmental Public Health Tracking Program, the CDC is working closely with state and local health departments to provide the resources and methods for assembling and presenting available hazard, exposure and health outcome data. This information will be made available to public health professionals, researchers and the public to improve the health and welfare of communities across the nation. The CDC's National Environmental Public Health Tracking Network (EPHTN), launched in July 2009, has a website that brings together environmental and health surveillance data [1].

One of the health indicators on the EPHTN is hospitalizations due to acute myocardial infarction (AMI), also known as heart attack. No national level surveillance exists for AMI (or coronary heart disease in general), except for mortality, in the US. Information on AMI incidence and prevalence has been limited to community studies [2]–[10], survey samples [11], and large cohort studies [12], [13]. Currently, AMI hospitalization counts and rates are available on the Tracking Network for more than 20 states (and their counties) beginning in 2000 [1]. The data on AMI hospitalizations has been collected using a consistent set of case definitions and includes all, not just a sample of, hospitalizations in the tracking states. This new data source provides the opportunity to examine time trends, assess geographic variation and demographic patterns, including differences by gender, and ultimately to evaluate primary and secondary prevention efforts.

Our objective in this study was to evaluate the temporal and spatial trends of acute myocardial infarction hospitalizations for all participating EPHTN states and counties over a nine year period (2000–2008).

## Methods

### Ethics

The University of Pittsburgh Institutional Review Board approved this research under an exempt classification. Data was de-identified and supplied by the CDC Environmental Public Health Tracking Network in an aggregate form for those 35 years and older per 10,000 population, stratified by gender.

### Data

The CDC provided calendar year-specific counts, crude rates, and age-adjusted rates for AMI hospitalizations between 2000 and 2008 for men and women aged 35 years and older for each state and county in the tracking network. Data were obtained from individual state hospital inpatient discharge record repositories and were provided to the CDC EPHTN by the environmental public health tracking program in each state. Included in this analysis were the 20 states submitting data to EPHTN: California, Colorado, Connecticut, Florida, Louisiana, Maine, Maryland, Massachusetts, Minnesota, Missouri, New Hampshire, New Jersey, New Mexico, New York, Oregon, Pennsylvania, South Carolina, Utah, Washington and Wisconsin.

The numerators for these rates were resident in-patient hospitalizations admitted during a calendar year with a primary discharge diagnosis code of 410.00–410.92 according to the International Classification of Diseases, Ninth Revision (ICD-9). Denominators were state- and county-specific mid-year population from the Census Bureau. CDC suppressed non-zero counts less than 6 and corresponding rates to protect confidentiality. CDC calculated age-adjusted rates using the direct method and the 2000 U.S. standard population, presented as per 10,000. County level rates included both unsmoothed rates and geographically smoothed rates. Geographic smoothing algorithms “borrow” information from neighboring areas to stabilize results from sparsely populated areas [14].

We calculated 95% confidence intervals (CI) for the age-adjusted rates using the following formula [15]:
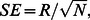
(1)where R is age-adjusted rate and N is number of events. The standard formula for determining the 95% CI of a rate is:
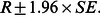
(2)The percentage change in AMI hospitalization age-adjusted rates was calculated from the first year of usable data and compared to the last year of usable data for each state:
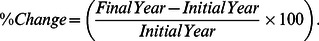
(3)We also calculated the ratio of male/female age-adjusted rates for each state by calendar year to compare gender disparity of AMI hospitalizations over time.

### Geographic Variation

To visualize geographic variations of AMI hospitalization rates, quantile maps of county-specific smoothed age-adjusted rates were graphed by calendar year using the quantile distribution for the year 2000 [16].

### Census Demographic Information and Behavioral Risk Factors

Census data at the state and county level for median income, percent below poverty level, percent high school grads among age 25+, percent with bachelor's degree, percent urban, and percent white and black from the 2000 census were obtained from the Census Bureau website: http://www.census.gov/main/www/cen2000.html. Correlation matrices were created to examine the ecological association between county level age-adjusted hospitalization rates for 2005 and the above demographic indicators.

Behavioral Risk Factor Surveillance System (BRFSS) data on current smoking, obesity (BMI>30), health care coverage, heavy drinking (>2 drinks/day for men; >1 drink/day for women), and vigorous physical activity (20+ minutes of vigorous physical activity 3 more days/week) was downloaded from the BRFSS website: http://www.cdc.gov/brfss/index.htm for each state for 2005.

### AMI Mortality

Mortality data were obtained from a compressed mortality file through CDC Wonder (http://wonder.cdc.gov/mortsql.html) provided by National Center for Health Statistics.

### Temporal Trend Analysis

To examine the overall temporal trend of AMI hospitalization rates, a longitudinal linear mixed effects model (LMEM) was fitted to the age-adjusted AMI hospitalization rates for 20 states and the counties within each state using S$ classes in R [17], [18]. This is a simple LMEM and assumes the relationship between AMI hospitalization rate and time is linear. The model allows for a random intercept and random slopes for states and counties. These random effects are assumed to be statistically independent of each other. The model is:
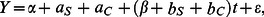
(4)where *Y* is the age-adjusted AMI hospitalization rate, *t* is the year minus 2000, *α* is the fixed effect intercept, *β* is the fixed effect slope, *a_s_* is the random intercept for states with variance 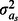, *a_c_* is the random intercept for county within state with mean 0 and variance 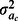, *b_s_* is the random slope for states with mean 0 and variance 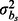, *b_c_* is the random slope for county within state with mean zero and variance 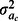, and 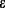 is a random error with mean zero and standard deviation σ. Based on these results for the fixed effects and random effects, predictions can be made for the intercepts (rates in 2000) and slopes for each state, and similarly, for each county.

## Results

Table 1 provides information on the distribution of the AMI hospitalization rates and population from 2000 to 2007 by gender and age group (35–64 and 65+) for the 20 CDC tracking states. There were 1,085,638 AMI hospitalizations among women and 1,404,360 AMI hospitalizations among men from 2000–2007. It should be noted that the 35–64 age group accounted for between 33.4% and 45% of the hospitalizations for women and men respectively, of the total AMI hospitalizations in these states during the time period.

**Table 1:** Distribution of AMI cases by gender and age group for CDC Tracking States: 2001–2007.

State	% AMI hospitalization by age group		Men		Women	
	35–64	≥65	Total pop*	No. of AMI cases	Total pop*	No. of AMI cases
California	37.8	62.2	141,064,890	289,637	141,551,592	188,161
Colorado §	45.0	55.0	9,502,945	15,677	9,354,083	8,728
Connecticut	34.8	65.2	13,453,148	34,519	14,228,647	25,325
Florida	37.1	62.0	67,162,112	168,387	69,985,922	101,509
Louisiana £	44.5	55.5	15,069,454	32,270	16,025,781	23,795
Maine Φ	37.6	62.4	4,450,533	19,546	4,678,227	13,787
Maryland	40.5	60.0	21,232,295	39,557	22,709,884	50,847
Massachusetts	33.4	66.6	24,857,636	56,924	26,556,309	75,942
Minnesota	37.7	62.3	20,098,544	46,199	20,375,023	30,225
Missouri	39.5	60.5	22,345,325	51,668	23,511,316	71,250
New Hampshire	40.9	59.1	5,052,845	14,401	5,205,201	9,005
New Jersey	36.2	63.8	33,418,676	98,006	35,172,359	69,803
New Mexico	42.2	57.8	7,424,688	15,134	7,644,051	9,349
New York	36.8	63.2	74,409,378	200,608	79,481,582	150,505
Oregon	37.1	62.9	14,184,472	33,563	14,392,220	21,474
Pennsylvania Φ	34.7	65.3	41,895,313	145,645	44,501,149	110,600
South Carolina	46.0	54.1	16,287,400	45,312	17,207,984	31,388
Utah	43.3	56.7	9,773,407	14,755	9,660,946	6,839
Washington	40.3	61.2	24,551,141	31,571	24,740,240	50,547
Wisconsin	36.0	64.0	21,760,396	50,981	22,145,331	36,559
Total			587,994,598	1,404,360	609,127,847	1,085,638

* total population for 2000–2007 based on census annual averages in states for whom data is available.

§ : AMI 2000–2003 N/A.

£ : AMI 2007 N/A.

^*Φ*^: AMI 2000 N/A.

### Temporal trends

Age-adjusted rates of AMI hospitalization by year and state are shown in Table 2 (2000–2004) and Table 3 (2005–2008) for men and women. Between 2000 and 2008, the age-adjusted rates for AMI hospitalization among men and women aged 35+ ranged from a low of 22.2/10,000 in Utah in 2008 to a high of 67.2/10,000 in Maine in 2001. There was an overall decrease in AMI hospitalization rates between 2000 and 2008 for all states, except for Florida, with most states showing over a 20% decrease during the period. For most states, this decrease represented a consistent decline over the time period. However, for a few states, the rates appear to level off or slightly increase since 2005 or 2006. The ratio of male/female rates for AMI hospitalization remained relatively consistent over time, approximately two-fold higher in men compared to women, and ranged from 1.65 to 2.66 within the EPHT states.

**Table 2:** Age-adjusted* rates of hospitalization for acute myocardial infarction (AMI) among persons 35 and over per 10,000 population, by state and year, 2000–2004.

State	2000 95% CI	2001 95% CI	2002 95% CI	2003 95% CI	2004 95% CI
California	**42.1** (41.8, 42.4)	**41.3** (40.9, 41.6)	**40.0** (39.6, 40.3)	**39.2** (38.9, 39.5)	**36.3** (36.0, 36.6)
Colorado	NA (NA, NA)	NA (NA, NA)	NA (NA, NA)	NA (NA, NA)	**30.4** (29.6, 31.1)
Connecticut	**44.5** (43.6, 45.5)	**41.4** (40.5, 42.3)	**40.9** (40.0, 41.8)	**40.5** (39.6, 41.4)	**37.9** (37.1, 38.8)
Florida	**32.8** (32.4, 33.1)	**32.6** (32.2, 32.9)	**32.2** (31.9, 32.6)	**31.3** (31.0, 31.7)	**29.7** (29.3, 30.0)
Louisiana	**37.4** (36.6, 38.2)	**39.3** (38.5, 40.2)	**41.4** (40.6, 42.3)	**38.2** (37.4, 39.0)	**37.9** (37.1, 38.7)
Maine	NA (NA, NA)	**67.2** (65.3, 69.0)	**65.7** (63.9, 67.6)	**65.8** (64.0, 67.6)	**57.5** (55.8, 59.2)
Maryland	**49.0** (48.1, 49.8)	**48.8** (47.9, 49.6)	**46.9** (46.1, 47.8)	**46.0** (45.2, 46.8)	**39.7** (38.9, 40.4)
Massachusetts	**50.9** (50.1, 51.6)	**51.1** (50.3, 51.8)	**50.9** (50.2, 51.7)	**51.1** (50.4, 51.9)	**45.6** (44.9, 46.3)
Minnesota	**42.7** (41.9, 43.5)	**42.3** (41.5, 43.1)	**40.6** (39.8, 41.4)	**37.9** (37.1, 38.6)	**35.8** (35.1, 36.5)
Missouri	**54.7** (53.9, 55.5)	**54.9** (54.0, 55.7)	**54.9** (54.1, 55.7)	**52.4** (51.6, 53.2)	**47.1** (46.3, 47.9)
New Hampshire	**50.4** (48.7, 52.2)	**48.8** (47.1, 50.5)	**45.4** (43.8, 47.1)	**45.6** (43.9, 47.2)	**40.8** (39.3, 42.3)
New Jersey	**51.1** (50.4, 51.7)	**49.7** (49.0, 50.3)	**50.2** (49.6, 50.9)	**47.7** (47.1, 48.4)	**43.8** (43.2, 44.4)
New Mexico	**35.7** (34.4, 36.9)	**35.0** (33.8, 36.2)	**32.8** (31.6, 33.9)	**31.7** (30.5, 32.8)	**30.7** (29.6, 31.8)
New York	**48.1** (47.7, 48.5)	**47.6** (47.2, 48.0)	**46.9** (46.5, 47.4)	**46.0** (45.6, 46.4)	**43.1** (42.7, 43.5)
Oregon	**38.4** (37.5, 39.3)	**38.2** (37.3, 39.0)	**39.2** (38.3, 40.1)	**37.5** (36.6, 38.3)	**33.8** (33.0, 34.6)
Pennsylvania	NA (NA, NA)	**54.4** (53.9, 54.9)	**53.3** (52.8, 53.9)	**52.0** (51.4, 52.5)	**48.0** (47.5, 48.5)
South Carolina	**50.5** (49.5, 51.5)	**49.4** (48.4, 50.3)	**47.7** (46.7, 48.6)	**45.5** (44.6, 46.4)	**41.3** (40.5, 42.2)
Utah	**38.0** (36.7, 39.4)	**34.9** (33.6, 36.2)	**34.8** (33.6, 36.1)	**31.2** (30.1, 32.4)	**27.9** (26.8, 29.0)
Washington	**37.8** (37.1, 38.5)	**36.5** (35.9, 37.2)	**37.0** (36.4, 37.7)	**34.5** (33.8, 35.1)	**32.5** (31.9, 33.2)
Wisconsin	**41.7** (41.0, 42.5)	**41.5** (40.7, 42.2)	**39.9** (39.1, 40.6)	**37.4** (36.7, 38.0)	**35.7** (35.0, 36.3)

* Rates were age-adjusted by the direct method to the 2000 US Standard population.

**Table 3:** Age-adjusted* rates of hospitalization for acute myocardial infarction (AMI) among persons 35 and over per 10,000 population, by state and year, 2005–2008.

State	2005 95% CI	2006 95% CI	2007 95% CI	2008 95% CI	% Change
California	**34.1** (33.8, 34.4)	**32.1** (31.8, 32.4)	**30.9** (30.6, 31.1)	NA (NA, NA)	−26.6%
Colorado	**28.3** (27.6, 29.0)	**27.7** (27.0, 28.4)	**25.4** (24.8, 26.1)	**24.5** (23.9, 25.2)	−19.4%
Connecticut	**34.2** (33.4, 35.0)	**30.7** (29.9, 31.5)	**32.4** (31.6, 33.2)	**34.5** (33.7, 35.3)	−22.5%
Florida	**28.4** (28.1, 28.7)	**29.1** (28.8, 29.4)	**29.7** (29.4, 30.1)	**35.3** (35.0, 35.7)	+7.6%
Louisiana	**30.7** (30.0, 31.4)	**30.5** (29.8, 31.3)	NA (NA, NA)	NA (NA, NA)	−18.4%
Maine	**58.1** (56.4, 59.8)	**55.9** (54.3, 57.5)	**53.7** (52.1, 55.3)	NA (NA, NA)	−20.1%
Maryland	**37.3** (36.6, 38.0)	**33.4** (32.7, 34.1)	**31.3** (30.6, 31.9)	**30.6** (30.0, 31.3)	−37.6%
Massachusetts	**42.2** (41.6, 42.9)	**40.1** (39.5, 40.8)	**37.8** (37.1, 38.4)	**35.7** (35.1, 36.3)	−29.9%
Minnesota	**33.2** (32.5, 33.9)	**30.9** (30.3, 31.6)	**28.5** (27.9, 29.1)	**27.8** (27.2, 28.4)	−34.9%
Missouri	**44.8** (44.0, 45.5)	**42.1** (41.4, 42.8)	**39.5** (38.8, 40.2)	**39.1** (38.5, 39.8)	−28.5%
New Hampshire	**38.7** (37.2, 40.1)	**37.0** (35.6, 38.4)	**37.6** (36.2, 39.0)	NA (NA, NA)	−25.4%
New Jersey	**40.6** (40.1, 41.2)	**38.7** (38.2, 39.3)	**37.0** (36.5, 37.6)	**36.6** (36.1, 37.1)	−28.4%
New Mexico	**27.7** (26.7, 28.8)	**31.9** (30.8, 32.9)	**30.2** (29.2, 31.3)	NA (NA, NA)	−15.4%
New York	**39.1** (38.7, 39.4)	**35.4** (35.1, 35.8)	**32.2** (31.9, 32.5)	**32.2** (31.9, 32.6)	−33.1%
Oregon	**32.2** (31.4, 33.0)	**31.9** (31.1, 32.6)	**30.8** (30.1, 31.6)	NA (NA, NA)	−19.8%
Pennsylvania	**44.8** (44.3, 45.2)	**42.8** (42.3, 43.2)	**39.5** (39.1, 40.0)	**39.7** (39.3, 40.1)	−27.0%
South Carolina	**40.2** (39.4, 41.0)	**38.9** (38.1, 39.7)	**37.1** (36.3, 37.8)	**36.0** (35.3, 36.8)	−28.7%
Utah	**25.1** (24.1, 26.1)	**25.1** (24.1, 26.1)	**22.3** (21.4, 23.2)	**22.2** (21.3, 23.1)	−41.6%
Washington	**30.9** (30.3, 31.5)	**29.3** (28.7, 29.8)	**28.4** (27.8, 29.0)	**27.3** (26.7, 27.8)	−27.8%
Wisconsin	**34.1** (33.4, 34.7)	**31.9** (31.2, 32.5)	**30.8** (30.1, 31.4)	**29.9** (29.3, 30.5)	−28.3%

* Rates were age-adjusted by the direct method to the 2000 US Standard population.

### Geographic Patterns

**Figure 1:**
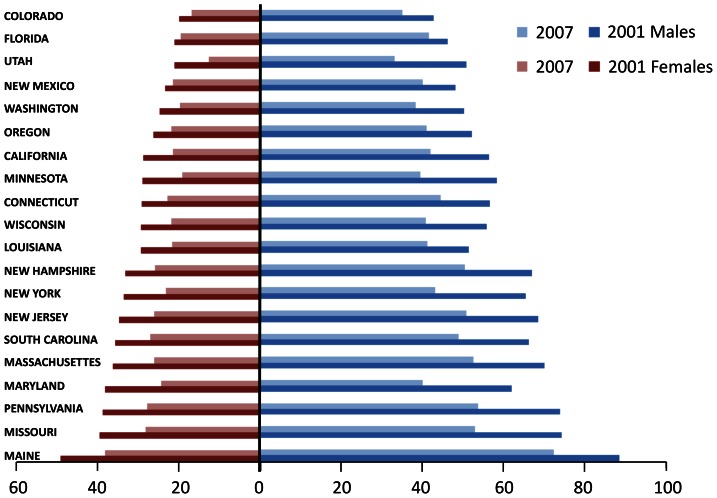
Age-adjusted hospitalization rates for acute myocardial infarction by gender and state, 2001 and 2007.

**Figure 2:**
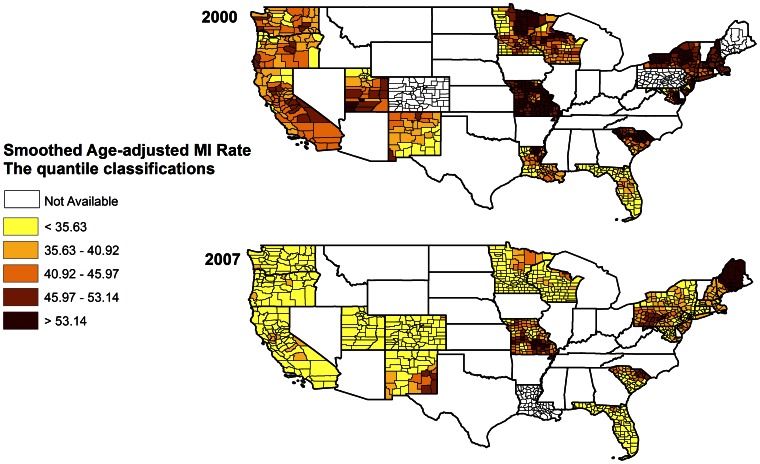
Smoothed age-adjusted rates of hospitalizations for AMI among persons 35 and over per 10,000 population by county.

Shown in Figure 1 are age adjusted AMI rates for men and women for 2001 and 2007. Large geographic variability was found for age-adjusted AMI hospitalization rates. It can be seen that in general, the highest rates were found in New England/Mid-Atlantic states. This is most evident in 2001 with the increase still evident in 2007 but somewhat narrowed. Seven of the nine highest states are in the New England/Mid-Atlantic corridor. The rates have been decreasing over time with the highest AMI rates showing the greatest reduction. Smoothed age adjusted rates for AMI at the county level for 2000 and 2007 are shown in Figure 2. Most of these rates have shifted to the first, second and third quantile based on the quantile classification in 2000.

### Predicted AMI Trends in LMEM

A longitudinal LMEM effects model was fitted using the age-adjusted AMI hospitalization rates from 929 counties within 20 states in the tracking network. The slope value of −1.8605 in fixed effects represents an average drop in AMI hospitalization rates across all states and the intercept value of 48.5124 represents the mean AMI hospitalization rate in 2000. The standard deviation of the residual error was 7.23. The average AMI hospitalization rate declined 3.8% annually. The slope is significantly different from zero at a p-value<0.0001, which indicates that there is a linear trend over time. The variance components indicate that the variation in the slopes and intercepts was relatively large for states as well as for counties within states. The intercept variance for state was 111.09 with an estimated standard deviation of 10.54, which indicates that each individual state would have a variation up to 10.54 or lower than the mean AMI hospitalization rate (Table 4).

**Table 4:** Linear mixed effects model typical, random effects, and state-specific intercepts and slopes for AMI hospitalization rates.

Typical (Fixed Effect)		
Level	Intercept	Slope
Country	48.51	−1.86
Random Effects Square Root of Variance Components		
State	10.54	0.74
Country	11.78	0.94

State-Specific					
State	Intercept	Slope	State	Intercept	Slope
California	42.03	−1.87	New Hampshire	53.83	−2.12
Colorado	39.12	−1.53	New Jersey	51.48	−1.95
Connecticut	46.63	−1.99	New Mexico	34.25	−0.38
Florida	31.36	0.20	New York	53.90	−2.35
Louisiana	46.18	−1.41	Oregon	45.22	−1.80
Maine	74.71	−2.45	Pennsylvania	63.67	−2.61
Maryland	53.10	−2.69	South Carolina	52.85	−1.87
Massachusetts	51.36	−1.89	Utah	40.12	−2.33
Minnesota	42.34	−2.12	Washington	42.03	−1.86
Missouri	61.41	−2.28	Wisconsin	44.66	−1.90

### Ecological analysis of Demographic and Behavioral Risk Factors

A county level analysis was conducted to consider sociodemographic factors and county level age adjusted hospitalization rates (Table 5). County level age-adjusted hospitalization rates were negatively correlated with median income, education (percent high school graduates), percent with a bachelor's degree, percent urban, and percent white and positively correlated with poverty (p<.01). The county census variables and yearly AMI hospitalization rates were also dichotomized by region (224 counties representing the Northeast and 706 counties representing the remaining tracking states.) Data not shown. The NE region counties had a higher median income, greater percent who graduated from high school, a lower percent black race and lower percent rural than the remaining 12 tracking state counties. Median income was $36,398 in non NE counties compared to $42,611 in NE counties (p<0.001), 79% completed high school compared to 82% in NE region (p<0.001), 6.3% of the counties' population in the NE were of the black race compared to 7.8% in the non NE (p<0.01) and percent rural was significantly lower (43% compared to 54%), (p<0.01). Multiple linear regression analysis was conducted with these four factors in the model as well as region. The NE region remained an independent predictor of AMI hospitalization rates for each year (2000–2008) (p<0.001). The results of both ecological analyses revealed that NE region remained significantly associated with AMI hospitalization rates after adjustment for sociodemographic factors.

**Table 5:** Sociodemographic factors by county and age adjusted AMI hospitalization rates 35+ years.

	Median Income	% Below poverty	% High School graduate (25+)	% With bachelor's degree (25+)	% urban	% white	% black
AMI Hospitalization Rate (2005)	−.237*	.096*	−.222*	−.306	−.229*	−.153*	−.028
Median Income	-------	−.736*	.630*	.730*	.490*	.113*	−.180*
Percent below poverty		-------	−.750*	−.422*	−.169*	−.591*	−.488*
Percent high school graduate			-------	.685*	.193*	.512*	−.492*
Percent with bachelor's degree				------------	.495*	−.001	−.107*
Percent urban					---------	−.290*	.130*
Percent white						---------	−.779*
Percent black							---------

* **p<0.01.**

In order to consider the relationship of behavioral risk factors such as smoking, physical activity, access to medical care and region, state BRFSS data for 2005 was used and applied to the 20 tracking states. Table 6 displays the association between the age-adjusted AMI hospitalization rates by state and current smoking, obesity, health care coverage, heavy drinking, and vigorous physical activity from BRFSS by state for 2005. We dichotomized region into New England/Mid-Atlantic versus all else.

**Table 6:** Correlation Matrix of state BRFSS data (2005) and AMI mortality, AMI hospitalization rate and Region.

	AMI mortality rate (2005)	Health care	Obese	Current smoker	Physical activity	Heavy drinker	Region
AMI hospitalization rate (2005)	0.398	0.399	0.183	0.368	−0.139	0.0868	0.614*
AMI mortality rate (2005)	-------	−0.163	0.782*	0.545**	−0.621*	−0.0001	0.016
Health care		-------	−0.278	−0.141	0.345	0.1043	0.539**
Obese			-------	0.538**	−0.637*	−0.0372	−0.244
Current smoker				-------	−0.660*	0.2009	0.003
Physical activity					-------	0.1574	0.018
Heavy drinker						-------	−0.085
Region							------

* **p<0.01.**

** **p<0.05.**

AMI hospitalization rates were positively associated with health care coverage (p = 0.07) and very significantly related to region (p<0.001). AMI age adjusted mortality rates were positively and significantly correlated with obesity, current smoking and negatively correlated with vigorous physical activity. AMI mortality was negatively correlated with health care coverage.

## Discussion

The most notable finding of this analysis was a significant decline in the AMI hospitalization rates within EPHT states from 2000 to 2008 with a preponderance of the higher rates across the time period for the Northeast/Mid-Atlantic corridor. Most states demonstrated a decline of over 20% during the period.

This decline is consistent with other recently published studies of temporal trends of AMI hospitalization rates in different populations in the United States. Fang et al, conducted a study of acute myocardial infarction hospitalization rates using a sample of inpatient records acquired from the National Hospital Discharge Survey, a national sample of hospitals. They determined hospitalization rates for acute myocardial infarction by age and gender from 1979 to 2005, aggregated by 3-year groupings. They found that the AMI hospitalization rate increased from 1979–1981 to 1985–1987, stabilized over the next decade, and then declined slowly after 1996 to 2003–2005 [19]. Chen et al. calculated annual AMI hospitalization rates from 2002 to 2007 among individuals who were Medicare enrollees and found a 5.8% annual decline after adjustment for age, sex, and race [12]. Yeh et al. reported a 24% relative decrease from 2000 to 2008 in AMI hospitalization rates among patients 30 years of age or older in a large integrated health care delivery system (Kaiser Permanente) in California [13]. Using the Health Care Cost Utilization Project (HCUP) data, a 20% sample of hospitalizations in 40 states, Wang et al. reported that the age adjusted rates for AMI hospitalizations for white men and women were reduced by 30.8 and 31.4% respectively during the period 2000 to 2007 [20]. In 2012, Yeh et al. investigated regional and temporal changes in Medicare AMI incidence for men and women 65 years and older. Over 32,000,000 million women were included in the population at risk annually for 2000–2008. Two stage hierarchical models were used to account for patient characteristics and state level random effects. Although MI incidence declined in all regions between 2000–2008, adjusted rates of annual decline varied by region from 2.9% to 6.2%. They noted a significantly lower rate for mountain and pacific coast states and a higher rate also for the eastern seaboard. There were widening disparities for MI incidence as measured by percent change of between state variance from 2000–2008. Significant declines in risk adjusted 30 day mortality were observed in all regions with the fastest declines observed in states with higher baseline mortality rates. The authors state that although geographic disparities may have increased, regional differences in associated mortality have narrowed [21].

Yeh et al. and others have noted that the reductions are most likely due to increasing emphasis on measures to reduce risk factors at the individual and community levels, including public bans on smoking and lower target levels of LDL cholesterol and blood pressure. The use of certain cardioprotective medications (statins, beta-blockers, and aspirin) have increased over time. They mention however that the countervailing trends in obesity and diabetes “may have an opposite effect over time” [13]. In addition, there has been a clear movement to tighten air pollution standards in the past ten years (PM_2.5_, ozone) as well as to introduce regulations for pollution controls for coal fired power plants and other industries. (http://www.epa.gov/compliance/resources/cases/civil/caa/coal/index.html). This has resulted in a measured decrease in PM_10_ and PM_2.5_ over time. Regional differences, however, remain in PM_2.5_ levels with the higher concentrations in the eastern US and California and lowest concentrations in central regions and the Northwest [22].

This descriptive analysis of AMI hospitalization rates from EPHT tracking states also showed geographic differences among states and within states. This geographic variation in AMI hospitalization rates may reflect true differences in disease rates representing differences in underlying risk factors or access to health care coverage. An ecological analysis of state and county rates and BRFSS and census data revealed a positive association between health care coverage and AMI hospitalization rates whereas there was a negative association with AMI mortality. While this finding appears counter intuitive, the hypothesis may be that having health care coverage may make it more likely that you do not hesitate to access care when you need it. The New England/Mid-Atlantic region had a higher overall proportion of the population who reported that they had health insurance yet they had the highest AMI hospitalization rate (p = .079). Some of the variation may reflect differences or changes in diagnostic techniques and criteria or coding practices for AMI.

There are also some limitations in this data that may result in differences in geographic areas. The following populations were not usually included: Veterans Affairs, Indian Health Services and prison populations and the percentages of these populations in a given area may affect rates. In addition, some hospital admissions for residents of a given state may not have been included if the hospitalization occurred out of state and notification of the admission was not provided by the state in which it occurred. Also, it should be noted that since the AMI hospitalization rates are based on events rather than individuals, multiple admissions for the same individual and illness event may be included in the data. Thus, an area (such as a county) with a higher percentage of hospital transfers for AMI may have higher AMI hospitalization rates than an area with a lower percentage of transfers. Information on transfers was collected for three states within the tracking network. It was noted that between 8.9 and 13.2% of admissions were identified in Maine, Maryland and Massachusetts as transfers and this affected the AMI hospital rate by 4.0 to 5.0% percent overall. The CDC tracking program is in the process of gathering this information on all 20 states and will be making this publically available. (Robert Knorr, DrPH chair, CDC Hospitalization working group, personal communication, December 20, 2012 ). This pattern suggests that at least for the Northeastern US region, transfers are not a likely explanation for the higher AMI hospitalization rates.

The strengths of this investigation are the population based nature of the CDC EPHTN database, the calculation of AMI hospitalization age adjusted rates for 35+ year old men and women, and the consistent methodology for data collection and convenient access of the data. Limitations include the exclusion of a race variable, lack of age specific rates and aggregated not individual level data.

Access to de-identified hospital AMI data at the state, county, and local level is valuable to enable state health departments and public health practitioners to assess primary and secondary prevention efforts. In addition, the data will likely prove useful to conduct research to explore novel relationships between health and environmental risk factors.

This is the first assessment of state and county level AMI hospitalization rates among individuals age 35 and over in the 20 states participating in the CDC tracking network, a surveillance system with a consistent methodology for data collection. We found a significant overall decrease in age adjusted AMI hospitalization rates from 2000 to 2008 with most states showing more than a 20% decline. In addition, there was substantial geographic variation in the rates. Continued population based surveillance of AMI hospitalizations and mortality is important. Expansion of the CDC EPHTN to include all states would permit a fuller picture of time trends, geographic differences, and populations at risk. The incorporation of BRFSS data on risk factors such as access to health care, smoking, and physical activity at the county level would also be of value to the ongoing surveillance of this disease. Further research using individual level data is needed to investigate geographic variations. Office outpatient records that can be linked to individual hospital records would create a valuable resource. Information on co-morbid conditions, medication and treatment history, and lifestyle information would add immeasurably to answering the complicated question surrounding spatial and temporal trends in AMI hospitalization and mortality.
